# The First Isolation and Characterization of Bat Jeilongviruses in Japan

**DOI:** 10.1155/tbed/5530007

**Published:** 2024-12-24

**Authors:** Sho Sata, Isshu Kojima, Mana Esaki, Kimitake Funakoshi, Masahiro Kajihara, Shinji Hirano, Shin Murakami, Kazuo Miyazaki, Makoto Ozawa, Kosuke Okuya

**Affiliations:** ^1^Joint Faculty of Veterinary Medicine, Kagoshima University, Kagoshima, Japan; ^2^Joint Graduate School of Veterinary Medicine, Kagoshima University, Kagoshima, Japan; ^3^Biological Laboratory, Faculty of Intercultural Studies, The International University of Kagoshima, Kagoshima, Japan; ^4^Division of International Research Promotion, International Institute for Zoonosis Control, Hokkaido University, Sapporo, Japan; ^5^Laboratory of Veterinary Microbiology, Graduate School of Agricultural and Life Sciences, The University of Tokyo, Tokyo, Japan; ^6^MiCAN Technologies Inc., Kyoto, Japan

## Abstract

Bats represent natural reservoirs of several paramyxoviruses, raising concerns about the potential for these viruses to cause cross-species infections. In this study, we isolated two jeilongviruses belonging to the family *Paramyxoviridae* from oral swab samples of the Eastern bent-wing bat (*Miniopterus fuliginosus*) and Far Eastern myotis bat (*Myotis bombinus*) in Kagoshima Prefecture, Japan. Notably, this is the first report isolating bat paramyxoviruses in Japan. Genomic analyses revealed a high identity between Kagoshima isolates (PMV/Bat35 and PMV/Bat111) and jeilongvirus B16-40, previously isolated from a Schreiber's bent-wing bat (*Miniopterus schreibersii*) in South Korea in 2016. PMV/Bat35 infected and replicated in a range of cell lines derived from different animal species, although the level of syncytium formation varied among cell lines. Animal experiments revealed that Syrian hamsters inoculated intranasally with PMV/Bat35 did not exhibit clinical symptoms or significant weight loss. Nevertheless, viral genes were detected in the lungs and tracheas of Syrian hamsters on 2- and 5-day postinfection (dpi). Importantly, neutralizing antibodies against PMV/Bat35 developed in hamsters on 14 dpi. These results suggest that bat jeilongviruses can cross the species barriers. Our findings highlight the critical importance of ongoing monitoring and characterization of viruses circulating in bat populations to assess the risk of zoonotic outbreaks.

## 1. Introduction

Bats are recognized as natural reservoirs for a wide range of viruses, such as coronaviruses and [1, 2]. This association between bats and viruses is likely driven by the unique ecological and physiological traits of bats, such as their ability to form large colonies, long lifespan, and flight, which facilitate virus maintenance and transmission [3]. Consequently, bats harbor a higher proportion of zoonotic viruses than other mammals [4]. In particular, bats frequently host viruses from the *Paramyxoviridae* family, some of which are causative agents affecting humans and livestock [2]. These characteristics highlight the need for further studies on bat–virus interactions to understand the mechanisms of viral spillover and potential zoonotic threats.

The *Paramyxoviridae* family consists of enveloped negative-stranded RNA viruses divided into nine subfamilies: *Avulavirinae*, *Feraresvirinae*, *Glossavirinae*, *Ichthysvirinae*, *Kamposvirinae*, *Metaparamyxovirinae*, *Orthoparamyxovirinae*, *Rubulavirinae*, and *Skoliovirina*. Paramyxoviruses have six structural proteins: nucleocapsid (N), phospho (P), matrix (M), fusion (F), attachment (H, HN, or G), and large (L) proteins [5, 6]. Recently, a number of novel bat paramyxoviruses were identified at different locations worldwide [7–11]. A new genus, *Jeilongvirus*, which belongs to the subfamily *Orthoparamyxovirinae*, has been designated and includes previously isolated viruses, such as the J virus [12]. Jeilongviruses are characterized by the presence of two additional proteins: a small hydrophobic (SH) protein and a transmembrane (TM) protein [13]. Although the hosts from which jeilongviruses have been isolated restricted to rodents and bats, recent surveillance using pan-paramyxovirus PCR and metagenomic approaches suggests that the host range extends beyond bats and rodents to include feline species [9, 14–18]. As of 2023, the International Committee on Taxonomy of Viruses (https://ictv.global/) recognizes 32 separate species of viruses belonging to the genus *Jeilongvirus*. However, despite extensive reports on the genetic diversity of this genus, the host specificity and pathogenicity of jeilongviruses remain unknown.

In this study, we isolated jeilongviruses from oral swabs samples collected from individuals of two microbat species, the eastern bent-wing bat (*Miniopterus fuliginosus*) and the Far Eastern myotis bat (*Myotis bombinus*), from distant sites in Kagoshima Prefecture, Japan. We genetically characterized the bat jeilongviruses and virologically evaluated their growth kinetics in a variety of cell lines. Furthermore, we elucidated the pathogenicity of one of the bat jeilongvirus isolates in Syrian hamsters.

## 2. Materials and Methods

### 2.1. Cells and Medium

African green monkey kidney-derived Vero, Vero cells stably expressing angiotensin-converting enzyme 2 derived from the Japanese little horseshoe bat (*Rhinolophus cornutus*) (Vero-RcACE2 [Rc] cells) [19], and baby hamster kidney BHK cells were maintained in Dulbecco's Modified Eagle's Medium (DMEM) (Fujifilm Wako Pure Chemical Corporation, Osaka, Japan) supplemented with 10% fetal bovine serum (FBS), 10,000 U/mL penicillin and 10 mg/mL streptomycin (PS) (Fujifilm Wako Pure Chemical Corporation) (10% FBS/DMEM). Vero E6 cells stably expressing human transmembrane serine protease 2 (VeroE6/TMPRSS2 cells) [20] were maintained in 10% FBS/DMEM supplemented with 100 µg/mL G418 (InvivoGen, San Diego, CA, USA). Calu-3 cells were maintained in Eagle's Minimum Essential Medium (EMEM) (Fujifilm Wako Pure Chemical Corporation) supplemented with 10% FBS and PS (10% FBS/EMEM). The PK-15 cells were maintained in 10% FBS/EMEM supplemented with a nonessential amino acid solution (NACALAI TESQUE, Kyoto, Japan). Madin–Darby canine kidney (MDCK) cells were maintained in minimum essential medium (MEM) (Thermo Fisher Scientific) supplemented with 5% newborn calf serum (NCS), 0.9 mM sodium bicarbonate, MEM amino acids (Thermo Fisher Scientific), MEM vitamin solution (Fujifilm Wako Pure Chemical Corporation), 2 mM L-glutamine, and PS. Human iPS cell-derived myeloid lineage iMylc-D05-s-ML2 cells (MiCAN Technologies, Kyoto, Japan) were maintained in a commercial medium (MiCAN Technologies). After inoculation with swab sample supernatant or bat jeilongvirus, the Vero, VeroE6/TMPRSS2, Rc, and BHK cells were maintained in 2% FBS/DMEM, whereas the other cells were maintained without any change in the relevant medium.

### 2.2. Sample Collection

**Figure 1:**
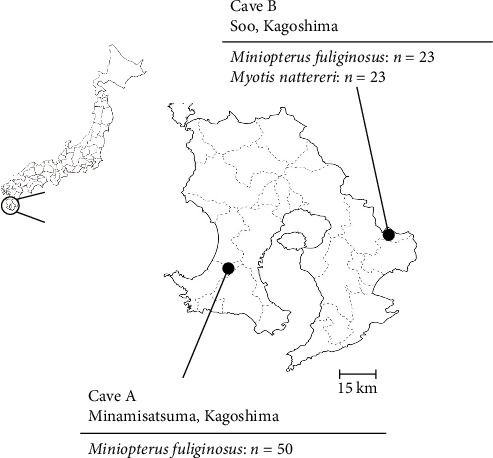
Sampling locations for collection of oral swabs from bats. The two caves are shown on a map of Kagoshima Prefecture, Japan, indicating the number of bats sampled per species.

To investigate viruses circulating among bat populations in Southern Japan, we collected oral swab samples using nylon flocked swabs (HydraFlock6, Puritan, MA, USA) from 50 and 46 microbats captured in Caves A and B, respectively, in Kagoshima Prefecture in 2022 (Figure 1). The bats were captured using butterfly nets and released immediately after the collection of oral swab samples. A permit for wildlife capture was obtained from the government of Kagoshima Prefecture (Permission number 155). The species of each bat was determined based on morphology. The swabs were suspended in laboratory-made transport medium (MEM supplemented with 0.5% bovine serum albumin [BSA], 2 mM L-glutamine, 0.3 mg/mL gentamicin, 2.5 μg/mL amphotericin B, and PS) and stored at 4°C or lower. The samples were immediately processed for virus isolation on the day of collection.

### 2.3. Virus Isolation

The suspended swab samples were centrifuged at 3000 rpm, 4°C, for 5 min and the supernatant was filtered using a 0.2-µm pore membrane (Sartorius, Göttingen, Germany). Subsequently, the supernatant was inoculated into the Vero, VeroE6/TMPRSS2, and Rc cells. After incubation for 1 h at 37°C, the inoculum was replaced with fresh medium. The samples were blindly passaged three times at 1-week intervals. The supernatant obtained from cells showing a syncytium formation was stored at ─80°C. Virus titers were determined using the median tissue culture infectious dose (TCID_50_) assay for each cell line.

### 2.4. Full Genome Sequencing

Supernatant obtained from cells infected with our isolates was filtered with a 0.2-µm pore membrane (Sartorius) and ultracentrifuged (Himac CS 120GX) at 24,000 rpm, 4°C, for 2 h with a sucrose cushion (15% sucrose in PBS). Nucleic acid in the precipitate was extracted using an innuPREP Virus DNA/RNA kit (IST Innuscreen GmbH, Reinach, Switzerland). Double-stranded cDNA (dscDNA) was synthesized from the total RNA using the PrimeScript Double Strand cDNA Synthesis Kit (TaKaRa Bio, Kusatsu, Japan). Subsequently, dscDNA was sequenced using a MinION Mk1B nanopore sequencer with a Flongle flowcell and a direct cDNA sequencing kit (SQK-DCS109) (Oxford Nanopore Technologies, Oxford, UK) or a reagent kit for sequencing (MiSeq Reagent Kit v3, Illumina, San Diego, CA, USA; 600 cycles). The obtained contigs were de novo assembled by the Geneious program with Medium Sensitivity/Fast using Geneious Prime (Dottmatics, Boston, MA, USA). Consensus sequences were subjected to BLAST analysis (https://blast.ncbi.nlm.nih.gov).

### 2.5. Phylogenetic Analyses

The nucleotide sequences of *Orthoparamyxovirinae* were retrieved from the NCBI nucleotide database (https://www.ncbi.nlm.nih.gov/nucleotide/) and aligned with those of our isolates using ClustalW [21] in MEGA7 software [22]. The aligned open reading frame of L, HN, and F genes was extracted and phylogenetic trees were constructed using the maximum-likelihood method with the general time-reversible plus gamma-distributed nucleotide substitution and invariant sites (GTR+G+I) model, which was suggested as the best-fit model for our datasets in MEGA7 software [22]. The robustness of each node was assessed using a bootstrap method (1000 replicates).

### 2.6. Quantification of Bat Jeilongvirus Using Real-Time Reverse Transcription (RT)-PCR

Real-time RT-PCR was performed using an iTaq Universal SYBR Green One-Step Kit (Bio-Rad, Hercules, CA, USA) in accordance with the manufacturer's instructions. The primer sets were designed based on the sequence of the L gene of our isolate, PMV/Bat35, to generate 140 bp PCR products (Bat-PMV16095F: GTTTACACCTGGGCCTTTGG; Bat-PMV16235R: TTTGCGTGCTCTTGTCTCTC). To generate standard calibration curves, the PCR product of the PMV/Bat35 L gene was inserted into a pCR Blunt II-TOPO cloning vector (Thermo Fisher Scientific).

PMV/Bat35 was inoculated into Rc, BHK, Calu-3, PK-15, and MDCK cells at multiplicity of infection (MOI) of 0.1 in Rc cells. After incubation for 1 h at 37°C, the inoculum was replaced with fresh medium. The supernatant was collected serially for up to 120 h postinfection (hpi). RNA was extracted using a MagMAX Viral/Pathogen Nucleic Acid Isolation Kit with KingFisher Duo Prime (Thermo Fisher Scientific, Waltham, MA, USA), in accordance with the manufacturer's instructions. The gene copy number was determined for each supernatant using real-time RT-PCR. Gene copy number was calculated as the mean of three independent trials.

### 2.7. Evaluation of Growth Kinetics From Virus Titer

PMV/Bat35 was inoculated into Vero, VeroE6/TMPRSS2, Rc, and iMylc-D05-s-ML2 cells at a MOI of 0.01 in each cell line. After incubation for 1 h at 37°C, the inoculum was replaced with fresh medium. The supernatants were serially collected up to 144 hpi. The viral titer of each supernatant was determined using TCID_50_ assays in Rc cells. Titers were calculated as the mean of three independent trials.

### 2.8. Experimental Infection of Hamsters

Three-week-old Syrian hamsters (*n* = 12) were purchased from Japan SLC Inc. (Hamamatsu, Japan) and housed in animal biosafety level-3 facilities at the Joint Faculty of Veterinary Medicine, Kagoshima University, Japan. Nine hamsters were inoculated with PMV/Bat35 and the remaining three hamsters inoculated with PBS were used as negative controls. Each of the PMV/Bat35-inoculated hamster received intranasal PMV/Bat35 at 7.12 × 10^4^ TCID_50_/200 µL, titrated in Rc cells. All of the hamsters were monitored for clinical signs and possible weight loss daily for up to 14 dpi. Among the virus-inoculated population, two hamsters were euthanized on both 2 and 5 dpi, and the following organs were immediately eviscerated: (brain, heart, lungs, trachea, stomach, intestine, colon, liver, spleen, and kidneys). Each collected organ was homogenized to prepare a 10% emulsion with laboratory-made transport medium using a TissueLyser II (QIAGEN, Hulsterweg, Netherlands). All the organ homogenates were stored at ─80°C until nucleic acid extraction. RNA was extracted from the organ homogenates using the MagMAX Viral/Pathogen Nucleic Acid Isolation Kit with KingFisher Duo Prime (Thermo Fisher Scientific) in accordance with the manufacturer's instructions, and the virus copy number was determined for each specimen using real-time RT-PCR (see above). All other PMV/Bat35-inoculated hamsters (*n* = 5) and negative control hamsters (*n* = 3) were euthanized on 14 dpi. Whole blood was obtained from the hamsters and centrifuged at 10,000 rpm, 4°C, for 5 min and the serum was collected for a microneutralization assay. The obtained serum was serially diluted (10 × 2^0^–10 × 2^12^) and mixed with PMV/Bat35 at 240 TCID_50_. After 1 h incubation, the serum–virus mixture was inoculated into Rc cells, which were then cultured for 5 days. All procedures involving Syrian hamsters were approved by the Institutional Animal Care and Use Committee of the Kagoshima University Experimental Animal Center (approval no. JFVM22049).

## 3. Results

### 3.1. Isolation of Paramyxovirus From Microbats in Kagoshima Prefecture

**Figure 2:**
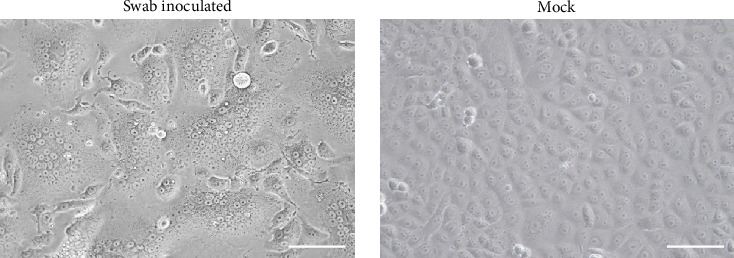
Isolation of paramyxovirus from a bat oral swab sample. Syncytium formation in Rc cells inoculated with PMV/Bat35 on 3 dpi. Scale bar: 100 μm.

A total of 96 oral swab samples were collected from individual microbats of two species (*Miniopterus fuliginosus*, *n* = 73; *M. bombinus*, *n* = 23) from two caves in Kagoshima Prefecture, Japan (Figure 1). To isolate any viruses, samples were inoculated into Vero and VeroE6/TMPRSS2 cells [20], and Rc cells [19]. Both VeroE6/TMPRSS2 and Rc cells are known to replicate bat coronaviruses related to severe acute respiratory syndrome coronavirus 2 (SARS-CoV-2) [19, 23]. Since no cytopathic effects were observed after inoculating the swab samples, the supernatant of the sample-inoculated cells was passaged blindly into the newly prepared cells. Three days after from blind passage, syncytium formation was observed in Rc cells inoculated with a sample collected from *Miniopterus fuliginosus* (Bat 35) at Cave A (Figures 1 and 2). Additionally, syncytium formation was observed in Vero cells inoculated with an oral swab sample from *M. bombinus* (Bat 111) collected at Cave B (Figure 1).

### 3.2. Genetic Analyses of PMV/Bat35 and PMV/Bat11 Isolates

**Figure 3:**
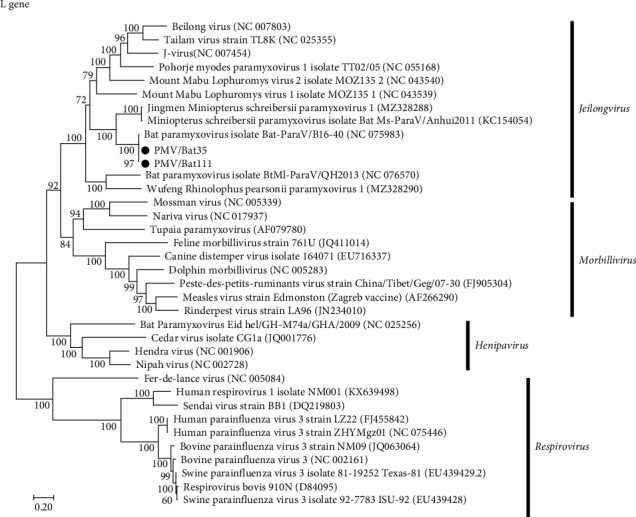
Phylogenetic analyses of genes derived from *Orthoparamyxovirinae*. Phylogenetic analyses were conducted based on the nucleotide sequences of L gene. The Kagoshima isolates, PMV/Bat35 and PMV/Bat111, are indicated in bold. Bootstrap values of >70% are shown at the nodes. The scale bar indicates the number of nucleotide substitutions per site.

Based on full genome sequencing, the viruses isolated from both Bat 35 and Bat 111 were classified as *Jeilongvirus miniopteri* (Table 1) and designated as PMV/Bat35 and PMV/Bat111, respectively. Genetic analyses revealed that genome sequences of our isolates, PMV/Bat35 and PMV/Bat111, were almost identical (>99.4% identity at the nucleotide sequence level) (Table 2). Furthermore, both PMV/Bat35 and PMV/Bat111 shared high genetic identity (>99.1% at the nucleotide sequence level) with bat jeilongvirus B16-40, which was isolated from a Schreiber's bent-wing bat (*Miniopterus schreibersii*) in South Korea in 2016 [9] (Table 3, Figure 3, and Supporting Information: Figure S1). Since our isolates showed high genetic identity, PMV/Bat35 was selected as a representative viral strain and used for the subsequent experiments, as described below.

### 3.3. Virus Replication in a Range of Cell Lines

**Figure 4:**
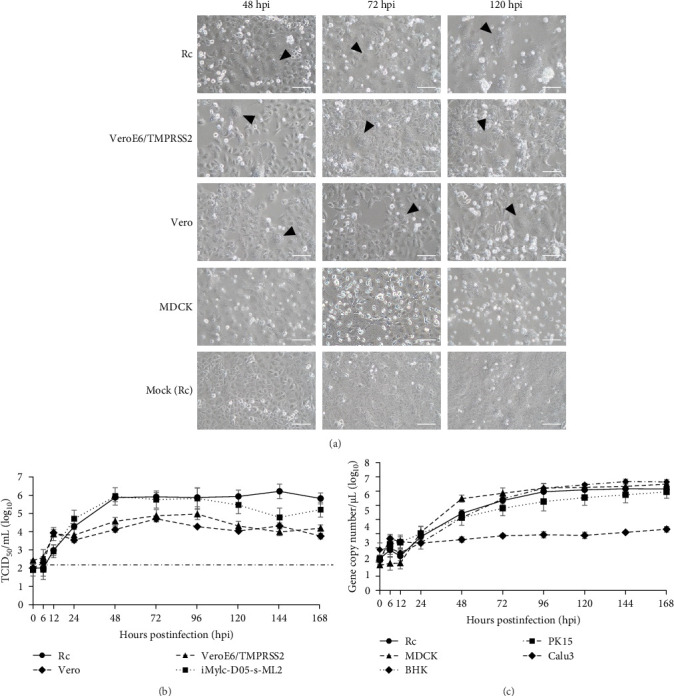
Virus growth kinetics in a range of cell lines. PMV/Bat35 has been inoculated into a range of cell lines derived from different animal species. Rc, VeroE6/TMPRSS2, Vero, and MDCK cells were observed under an optical microscope at 48, 72, and 120 hpi. Scale bar: 100 μm (A). The viral titer of PMV/Bat35 was determined at several time points in Rc, VeroE6/TMPRSS2, Vero, and iMylc-D05-s-ML2 cells using TCID_50_ assays. The dotted line indicates the detection limit (B). The gene copy number of PMV/Bat35 was determined at specific time points in Rc, MDCK, BHK, PK-15, and Calu-3 cells using real-time RT-PCR (C).

To identify animal species susceptible to infection by bat jeilongviruses, cell lines derived from different animal species were inoculated with PMV/Bat35. In the preliminary experiments, the infectivity of PMV/Bat35 in these cell lines was assessed based on syncytium formation. Syncytium formation was observed in VeroE6/TMPRSS2, Rc, and iMylc-D05-s-ML2 cells by 48 hpi (Figure 4A), but not in BHK cells, human lung cancer-derived Calu-3 cells, or porcine kidney-derived PK-15 cells (Supporting Information: Figure S2). Furthermore, PMV/Bat35-inoculated MDCK cells did not show syncytium formation, although they gradually detached by 120 hpi (Figure 4A).

To further analyze the growth kinetics of PMV/Bat35 in each cell line, we collected supernatants from cells infected with PMV/Bat35 at several time points and measured the viral titers. For cell lines exhibiting syncytium formation after PMV/Bat35 infection—Vero, VeroE6/TMPRSS2, Rc, and iMylc-D05-s-ML2 cells—TCID_50_ was calculated (Figure 4B). In all four cell lines, the PMV/Bat35 titer began to increase at 12 hpi and reached a plateau at ~72 hpi (Figure 4B). Notably, PMV/Bat35 efficiently replicated in Rc and iMylc-D05-s-ML2 cells, achieving titers higher than 10^5^ TCID_50_/mL.

For the cell lines that did not display syncytium formation—MDCK, BHK, Calu-3, and PK-15 cells—, the viral gene copy numbers in the supernatant were measured (Figure 4C). To confirm that viral gene copy numbers correlated with virus titers, we measured the viral gene copy numbers in Rc cells. Intriguingly, we found that the copy number of PMV/Bat35 increased without syncytium formation in PK-15, MDCK, and BHK cells (Figure 4C). Remarkably, this increase in the viral gene copy number was comparable to that observed in Rc cells (Figure 4B,C). Notably, the gene copy number in the Calu-3 cells increased slightly throughout the experimental period (Figure 4C). These results suggest that bat jeilongviruses have the potential to infect a wide range of animal species, including humans.

### 3.4. Infectivity of PMV/Bat35 in Hamsters

**Figure 5:**
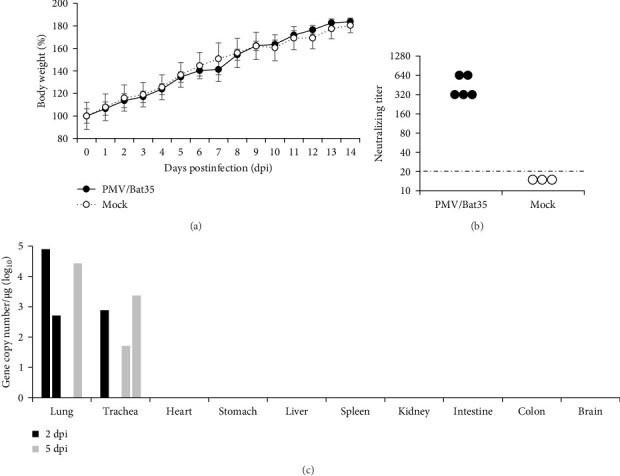
Infectivity of PMV/Bat35 in hamsters. Syrian hamsters were inoculated intranasally with PMV/Bat35, observed for clinical signs, and subjected to daily body weight measurement until 14 dpi (A). The hamsters were euthanized on 14 dpi and serum was collected for serum neutralization assays. The dotted line indicates the detection limit (B). Four hamsters were euthanized on 2 dpi (*n* = 2) or 5 dpi (*n* = 2), and their major organs (brain, lungs, trachea, heart, stomach, intestine, colon, liver, spleen, and kidneys) were collected. The gene copy number in major organ homogenates was determined using real-time RT-PCR (C).

Syrian hamsters are an animal model for a broad range of paramyxoviruses, including the genera *Henipavirus*, *Moribillivirus*, and *Avulavirus* [24–26]. To investigate the pathogenicity of our bat jeilongvirus, Syrian hamsters were intranasally inoculated with PMV/Bat35. The hamsters did not show clinical symptoms or marked weight loss during the experimental period of 14 days postinfection (dpi) (Figure 5A). However, neutralizing antibodies were detected in serum samples collected from hamsters on 14 dpi (Figure 5B), indicating that the hamsters were infected with PMV/Bat35. Furthermore, viral genes were detected in the lungs and tracheas collected on 2 and 5 dpi, respectively (Figure 5C). These results suggest that bat jeilongviruses replicate in the respiratory tract of other animal species without causing any clinical symptoms.

## 4. Discussion

In this study, we isolated bat jeilongviruses from oral swab samples, obtained from *Miniopterus fuliginosus* and *M. bombinus*, in different caves in Kagoshima Prefecture, Japan (Table 1). Notably, this is the first report of the isolation of a bat paramyxovirus in Japan. *M. bombinus* often forms maternity colonies with *Miniopterus fuliginosus* in Southwestern Japan [27]. Indeed, these two species formed mixed colonies in Cave B (Figure 1). *Miniopterus fuliginosus* also forms mixed colonies with *Myotis macrodactylus* and *Rhinolophus ferrumequinum* in Kyushu [27], suggesting that multiple bat species are potential reservoirs of bat jeilongviruses.

Genome sequencing revealed that the two bat jeilongviruses, PMV/Bat35 and PMV/Bat111, were genetically almost identical to jeilongvirus B16-40, isolated from *Miniopterus schreibersii* in South Korea in 2016 [9] (Figure 3, Supporting Information: Figure S1, and Table 3). The high aspect ratio of the *Miniopterus schreibersii* wings suggests that it is adapted to cover long distances and is capable of traveling over 200 km [28, 29]. Thus, bat jeilongviruses may have crossed the border between Japan and South Korea via transmarine flights of *Miniopterus schreibersii* and/or *Miniopterus fuliginosus*.

We found that PMV/Bat35 replicated in cell lines derived from a range of animal species (Figure 4), suggesting the potential of bat jeilongviruses to cross species barriers. Many paramyxoviruses, including human parainfluenza viruses 1 and 3, Newcastle disease virus, and the mumps virus, utilize sialic acid as a receptor for cell binding [30–32]. Notably, jeilongvirus B16-40 utilizes sialic acid to bind to cells [33]. Although viral replication depends on other host factors beyond the presence of sialic acid, the highly conserved structure of sialic acid in mammals may allow viruses to cross the species barriers [34]. It is interesting to note that while jeilongviruses in early reports were mainly detected in rodents and bats [11, 12], recent articles have reported their detection in hedgehogs and cats [16, 35]. Furthermore, antibodies against another jeilongvirus, the J virus, which was originally isolated in mice, have been found in swine, cattle, and humans [12]. Taken together, our findings suggest that bat jeilongviruses have a broad range of hosts.

Syncytium formation is a common cytopathic effect observed in paramyxovirus infections [36]. However, PMV/Bat35 did not induce syncytium formation in any of the evaluated cell lines. Such variability in syncytium formation has also been reported in other paramyxoviruses. For instance, a reduction in cellular cholesterol leads to a dose-dependent decrease in syncytium formation caused by Nipah virus infection [37]. Furthermore, various cell–cell fusion events are associated with phosphatidylserine (PS) exposure on the cell surface [38–42]. Phospholipid scramblases catalyze PS exposure on cell surfaces [43]. Therefore, the variability in syncytium formation may also be attributed to differences in the levels of cellular cholesterol and PS, as well as the activity of host cell phospholipid scramblases.

To further investigate the infectivity and pathogenicity of bat jeilongviruses, Syrian hamsters were intranasally inoculated with the PMV/Bat35 isolate. Syrian hamsters were chosen as the animal model for this study because of their widespread use in research on various paramyxoviruses, including the Hendra virus, Nipah virus, respiratory syncytial virus, and human parainfluenza virus [44–47]. Importantly, a previous study showed that C57BL/6 mice are not infected with the bat jeilongvirus B16-40 [9]. In this study, hamsters were asymptomatically infected with PMV/Bat35 (Figure 5), with viral replication in the lungs and tracheas (Figure 5C). These results highlight the complexity of viral host range, as viral replication is influenced by various factors beyond just the presence of a receptor, such as host immune responses and intracellular compatibility. Since our isolates were obtained from oral swab samples, we believe that this virus may also replicate in the respiratory tract of microbats. Our findings suggest that bat jeilongviruses have the potential to cross the species barrier without causing any marked symptoms.

## 5. Conclusions

We isolated jeilongviruses from microbats in the Kagoshima Prefecture, Japan. We also found that jeilongviruses have the potential to infect other animal species without causing clinical symptoms. This study highlights the importance of monitoring and characterizing bat paramyxoviruses to assess the potential risk of zoonotic outbreaks.

## Figures and Tables

**Table 1:** Bat jeilongviruses isolated in this study.

Virus	Collected date	Location	Bat species	Sex	Young/adult	Genbank
PMV/Bat35	January 28, 2022	Cave A	*Miniopterus fuliginosus*	N/A	N/A	PP990581
PMV/Bat111	April 19, 2022	Cave B	*M. bombinus*	**♀**	Adult	PP990582

Abbreviation: N/A, not available.

**Table 2:** Identity at the nucleotide and amino acid sequence level for PMV/Bat35 and PMV/Bat111.

Gene	Nucleotide sequence (%)	Amino acid sequence (%)
N	99.53	99.80
P	99.67	99.80
M	99.81	100.0
F	99.52	99.64
SH	99.72	99.58
TM	99.41	98.93
HN	99.66	99.49
L	99.79	99.95

**Table 3:** Identity at the nucleotide and amino acid sequence level between both PMV/Bat35 and PMV/Bat111, and Bat-ParaV/B16-40.

Gene	Isolate	Bat-ParaV/B16-40	
		Nucleotide sequence (%)	Amino acid sequence (%)
N	PMV//Bat35	99.53	99.80
	PMV/Bat111	99.47	100

P	PMV//Bat35	99.60	99.80
	PMV/Bat111	99.53	99.60

M	PMV//Bat35	99.24	100
	PMV/Bat111	99.24	100

F	PMV//Bat35	99.15	99.27
	PMV/Bat111	99.64	99.64

SH	PMV//Bat35	99.31	98.33
	PMV/Bat111	99.31	98.75

TM	PMV//Bat35	99.17	98.58
	PMV/Bat111	99.17	98.58

HN	PMV//Bat35	99.43	99.15
	PMV/Bat111	99.32	98.98

L	PMV//Bat35	99.51	99.86
	PMV/Bat111	99.45	99.91

## Data Availability

The data used to support the findings of this study are included within the article and in the supporting files.

## Supporting Information

Supporting Information Figure S1: Phylogenetic analyses of genes derived from *Orthoparamyxovirinae*. Phylogenetic analyses were conducted based on the nucleotide sequences of F (A) and HN (B). The Kagoshima isolates, PMV/Bat35 and PMV/Bat111, are indicated in bold. Bootstrap values of >70% are shown at the nodes. The scale bar indicates the number of nucleotide substitutions per site. Figure S2. Cells inoculated with PMV/Bat35. Rc cells, PK-15 cells, BHK cells, and Calu-3 cells inoculated with PMV/Bat35 were observed under an optical microscope at 0, 48, 72, and 120 hpi. Scale bar: 100 μm.
