# Supplementary material for: The First Isolation and Characterization of Bat Jeilongviruses in Japan
Source: Transbound Emerg Dis. 2024 Dec 24;2024:5530007. doi: 10.1155/tbed/5530007 (PMC12017206; doi:10.1155/tbed/5530007)
Supplement: Supporting Information — Figure S1: Phylogenetic analyses of genes derived from Orthoparamyxovirinae. Phylogenetic analyses were conducted based on the nucleotide sequences of F (A) and HN (B). The Kagoshima isolates, PMV/Bat35 and PMV/Bat111, are indicated in bold. Bootstrap values of >70% are shown at the nodes. The scale bar indicates the number of nucleotide substitutions per site. Figure S2. Cells inoculated with PMV/Bat35. Rc cells, PK-15 cells, BHK cells, and Calu-3 cells inoculated with PMV/Bat35 were observed under an optical microscope at 0, 48, 72, and 120 hpi. Scale bar: 100 μm. [file 5530007.f1.pdf]

(A) F gene

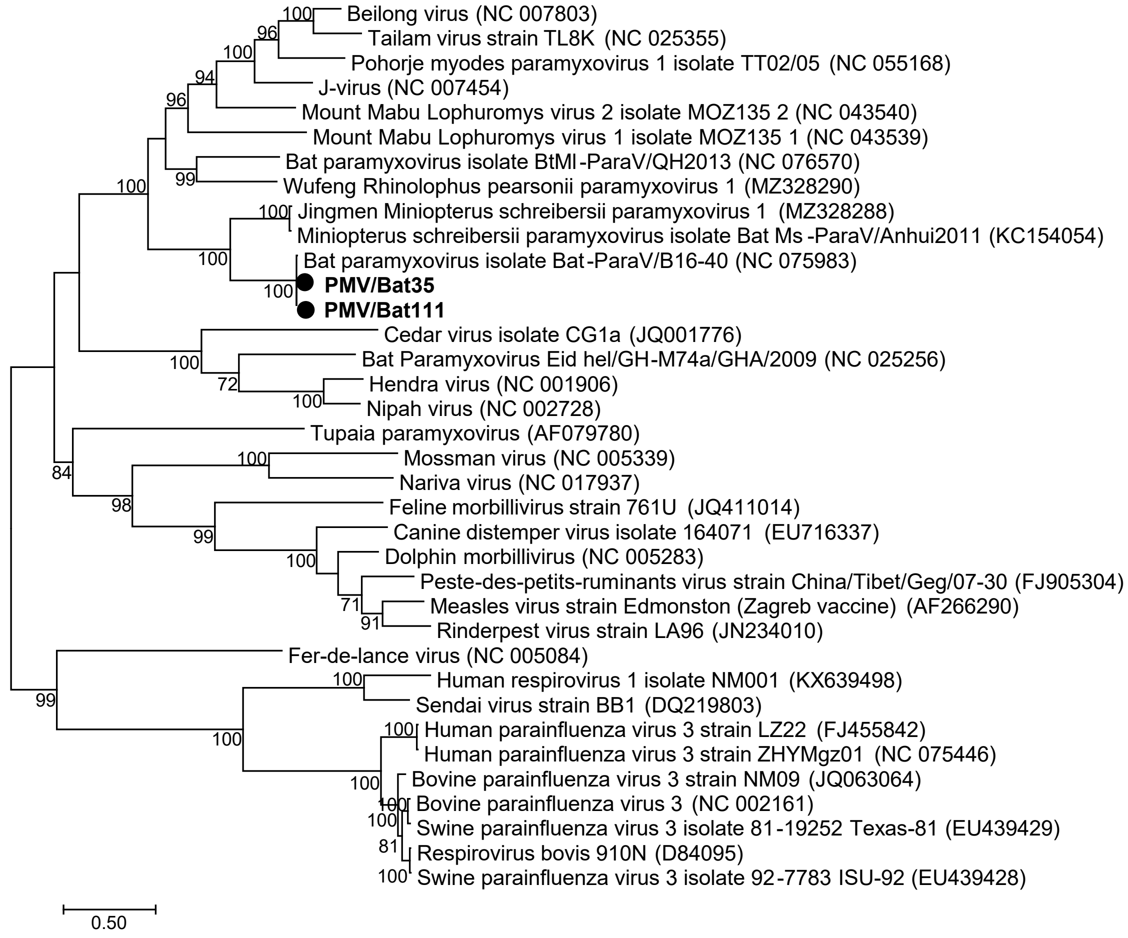

Supplementary Figure S1A. Phylogenetic analyses of genes derived from *Orthoparamyxovirinae*
Phylogenetic analyses were conducted based on the nucleotide sequences of F gene. The Kagoshima isolates, PMV/Bat35 and PMV/Bat111, are indicated in bold. Bootstrap values of >70% are shown at the nodes. The scale bar indicates the number of nucleotide substitutions per site.

(B) HN gene

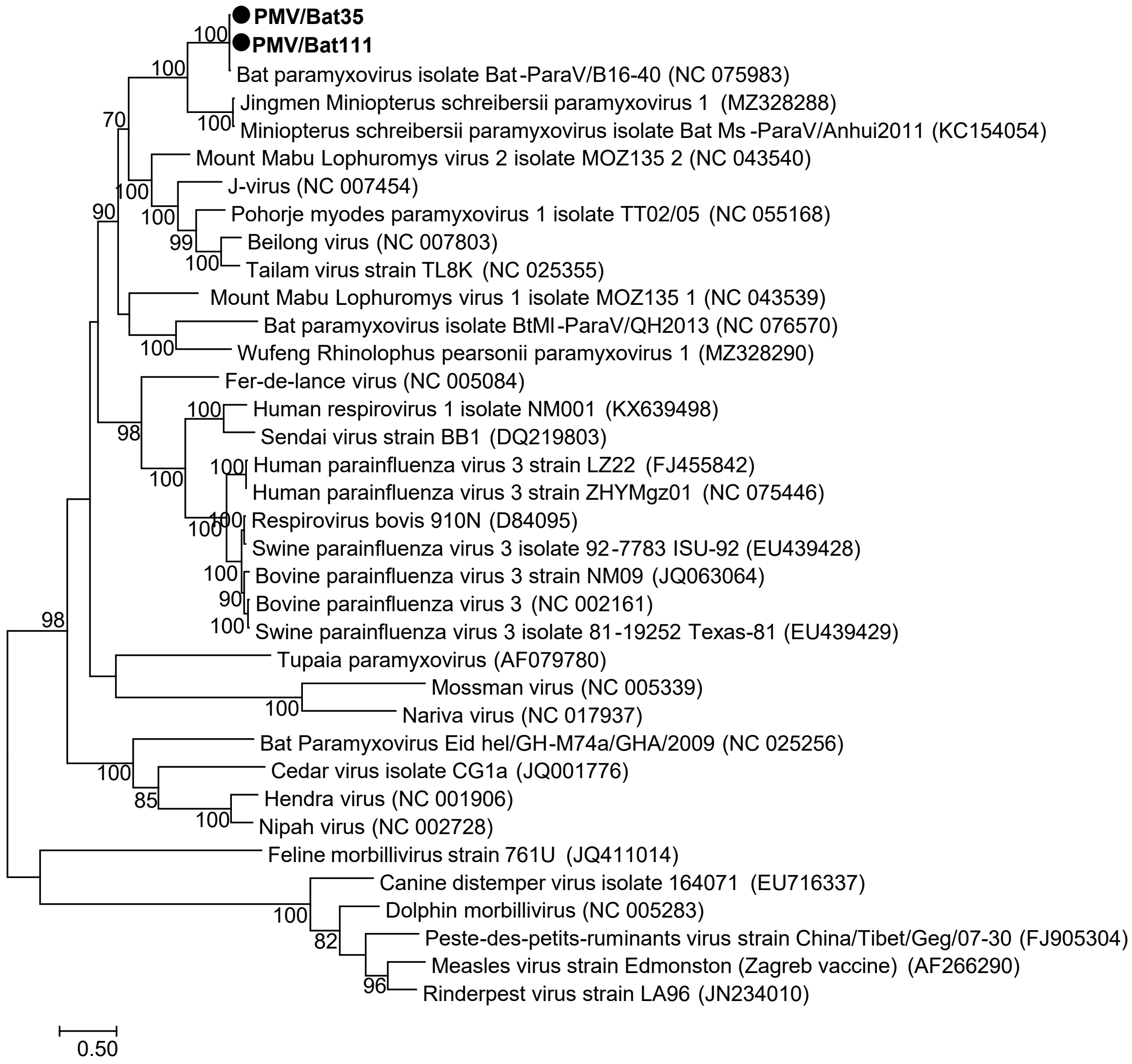

Supplementary Figure S1B. Phylogenetic analyses of genes derived from *Orthoparamyxovirinae*
Phylogenetic analyses were conducted based on the nucleotide sequences of HN gene. The Kagoshima isolates, PMV/Bat35 and PMV/Bat111, are indicated in bold. Bootstrap values of >70% are shown at the nodes. The scale bar indicates the number of nucleotide substitutions per site.

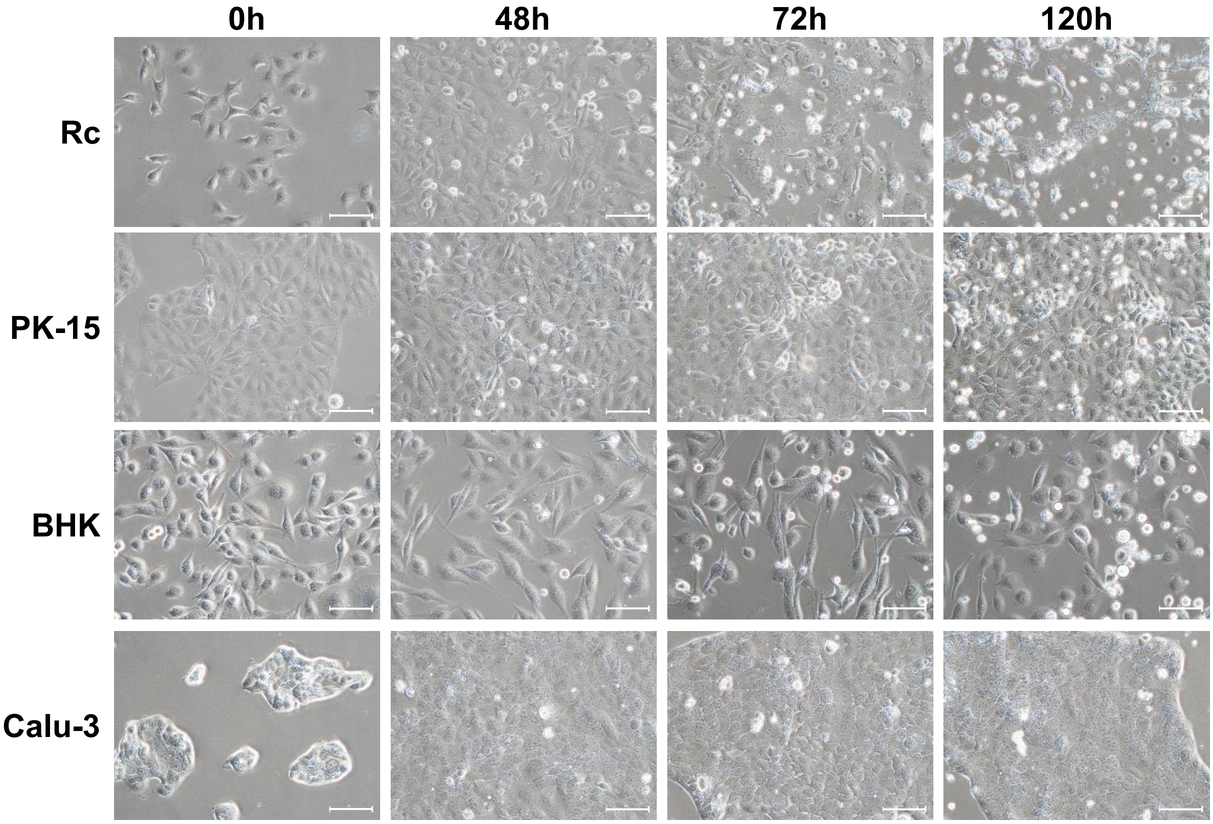

Supplementary Figure S2. Cells inoculated with PMV/Bat35

Rc cells, PK-15 cells, BHK cells and Calu-3 cells inoculated with PMV/Bat35 were observed under an optical microscope at 0, 48, 72, and 120 hpi. Scale bar: 100 μm
